# Oxidative Stress in Cataract Formation: Is There a Treatment Approach on the Horizon?

**DOI:** 10.3390/antiox13101249

**Published:** 2024-10-16

**Authors:** Jingyan Li, Francesco Buonfiglio, Ying Zeng, Norbert Pfeiffer, Adrian Gericke

**Affiliations:** Department of Ophthalmology, University Medical Center, Johannes Gutenberg University Mainz, Langenbeckstrasse 1, 55131 Mainz, Germany; fbuonfig@uni-mainz.de (F.B.); yingzeng46@gmail.com (Y.Z.); norbert.pfeiffer@unimedizin-mainz.de (N.P.)

**Keywords:** cataract, oxidative stress, aging, hypertension, diabetes, novel treatment approaches, antioxidants

## Abstract

Cataracts, a leading cause of blindness worldwide, are closely linked to oxidative stress-induced damage to lens epithelial cells (LECs). Key factors contributing to cataract formation include aging, arterial hypertension, and diabetes mellitus. Given the high global prevalence of cataracts, the burden of cataract-related visual impairment is substantial, highlighting the need for pharmacological strategies to supplement surgical interventions. Understanding the molecular pathways involved in oxidative stress during cataract development may offer valuable insights for designing novel therapeutic approaches. This review explores the role of oxidative stress in cataract formation, focusing on critical mechanisms, such as mitochondrial dysfunction, endoplasmic reticulum stress, loss of gap junctions, and various cell death pathways in LECs. Additionally, we discuss emerging therapeutic strategies and potential targeting options, including antioxidant-based treatments.

## 1. Introduction

Lens opacification, commonly referred to as cataract, is one of the leading causes of irreversible visual loss worldwide, with surgery being the only available treatment [1,2,3]. A recent meta-analysis involving 161,947 participants estimated the global cross-age prevalence of cataracts at 17%, with a strong correlation to age. Cataract prevalence was reported at 3% for individuals aged 20 to 39 years, rising to 54% in those over 60 years old [4]. Among the 33.6 million adults aged 50 and older who were blind in 2020, cataracts were the leading cause of blindness, affecting approximately 15.2 million people worldwide [5]. Given the high prevalence and severity of this condition, cataracts have become a critical public health issue, imposing significant societal burdens on a global scale [6]. These challenges highlight the urgent need for innovative therapeutic strategies to address the global shortfall in surgical availability [7]. This underscores the importance of developing methods to delay or prevent cataract formation.

Common symptoms of cataracts include impaired vision, reduced contrast sensitivity, color distortion, and increased sensitivity to glare [8]. The process of cataract formation remains a subject of debate due to its multifactorial nature [9]. Key risk factors include aging, systemic hypertension, diabetes, and ultraviolet (UV) light exposure [10,11,12]. All of these factors contribute to the generation of reactive oxygen species (ROS) in the lens, which play a critical role in cataract formation [13].

The aim of this work is to provide a comprehensive and updated overview of the redox mechanisms involved in cataract formation, with a particular focus on age-related, hypertensive, and diabetic cataracts, emphasizing their connection to oxidative stress. Furthermore, we review the latest research on the potential of antioxidants in preventing or slowing the progression of cataracts.

## 2. Anatomy, Composition, and Function of the Lens

The lens is a transparent, refractive, biconvex structure in the eye, primarily composed of epithelium and fibers [14]. Located behind the iris, it plays a critical role in the optical system, dynamically focusing images onto the retina [8]. In adults, the lens is encapsulated and features a monolayer of cuboidal lens epithelial cells (LECs) on its anterior surface. These LECs undergo differentiation at the lens equator, elongating and losing their organelles to form layers of lens fiber cells [15]. LECs are essential for maintaining the internal stability and transparency of the lens [16]. As the primary barrier protecting the crystalline lens from harmful external factors, LECs are crucial in preventing age-related cataract (ARC); when these cells become dysfunctional, cataract formation is initiated [17,18]. A detailed understanding of lens structure and function is critical, as disruptions in these processes can lead to pathological conditions, like cataracts. Cataract development is closely linked to the gradual accumulation of oxidative damage in the long-lived crystallin proteins of the lens, which leads to protein aggregation, reduced transparency, and, ultimately, cataract formation [16,19].

Crystallins in the human lens are broadly categorized into three groups, *α*-, β-, and γ-crystallins [20]. These proteins make up approximately 90% of the water-soluble proteins in the human lens, with α-crystallin alone accounting for around 40% [21]. However, mutations or post-translational modifications in α-crystallins, such as oxidation, deamidation, truncation, or crosslinking, can impair their function and contribute to disease progression [22]. Prolonged exposure to UV and visible light from solar radiation can increase hydrophobic exposure in αA-crystallin, alter its secondary structure, and reduce its chaperone activity [23]. Zhu et al. identified a higher percentage of the D-Asp 58 isomer in αA-crystallin in the lens cortex of diabetic patients with cataracts compared to individuals experiencing normal aging [24].

Among the three main vertebrate subtypes, β-crystallins exhibit the greatest range of polydispersity due to their complex multimerization characteristics in their natural state [25]. Both β- and γ-crystallins are extremely stable proteins in the vertebrate lens, evolved to reduce light scattering and enhance the refractive index, thus preserving lens transparency [26]. As the lens ages, crystallins undergo several post-translational modifications, including phosphorylation, glycation, and oxidation, which can lead to protein unfolding, aggregation, and precipitation [27,28].

## 3. Redox Homeostasis and Its Relevance in the Lens

Oxidative stress plays a critical role in the molecular mechanisms leading to cataract development [1,29]. Common pathological conditions, such as arterial hypertension and diabetes mellitus, are major drivers of cataractogenesis through the overproduction of ROS [30,31,32,33,34,35,36]. Additionally, the anterior segment of the eye is highly exposed to UV radiation, making UV exposure one of the most significant environmental sources of oxidative stress [37]. Consequently, blocking ROS generation and removing excess ROS through various pathways have been identified as potential therapeutic strategies for managing several eye disorders [38,39,40,41,42,43,44].

Disruption of redox homeostasis occurs when the body produces excessive ROS that cannot be adequately neutralized by antioxidant defenses [45,46,47,48,49,50]. ROS include both radical species, such as superoxide anion (O_2•_^−^), and non-radical molecules, like hydrogen peroxide (H_2_O_2_), which are highly reactive by-products of cellular metabolism produced during normal physiological processes, in response to pathological conditions, or due to environmental exposures [51,52,53]. Under normal conditions, ROS are generated as part of essential cellular functions, such as energy production, metabolism, and responses to infection, inflammation, or hypoxia [54]. External harmful agents, like radiation, smoking, toxins, and alcohol, can also stimulate ROS production [45,55]. Additionally, reactive chlorine species (e.g., hypochlorous acid, HClO) and reactive nitrogen species (RNS), such as peroxynitrite (ONOO−), are also considered part of the ROS family [56,57,58,59].

When an excess of ROS and RNS disrupts the redox balance, it leads to the degradation of vital biomolecules, including proteins, lipids, and DNA, compromising cellular integrity and function and ultimately resulting in cell death [54]. These reactive species can interact directly with cellular components, causing inflammation, accelerating aging processes, and eventually leading to cellular dysfunction and death [46,50,60].

### 3.1. Endogenous Antioxidants in the Lens

#### 3.1.1. The Glutathione System

The lens is equipped with a robust antioxidant defense system, featuring high levels of the potent antioxidant glutathione (GSH) [61]. GSH plays multiple roles in regulating redox homeostasis by serving as a carrier and reservoir for cysteine, as well as detoxifying aldehydes [62]. GSH homeostasis in lens cells is primarily maintained through its synthesis by LECs and outer fiber cells [10]. Additionally, a minor contribution to GSH levels comes from the intake of GSH/oxidized glutathione (GSSG) from the surrounding ocular environment [63]. Once synthesized, GSH is distributed throughout the lens and can be exported into the ocular environment [64,65]. GSH neutralizes H_2_O_2_ by converting it into GSSG disulfide, which is subsequently reduced back to GSH by the enzyme glutathione reductase (GR) in conjunction with nicotinamide adenine dinucleotide phosphate (NADPH) [66].

As the lens ages, GSH levels, particularly in the center of the lens, decline, leading to biochemical changes that cause protein aggregation, light scattering, and the development of age-related nuclear cataracts (ARNC) [67]. Studies by Carey and colleagues revealed that depletion of GSH in LECs reduces resistance to oxidative stress-induced damage, contributing to cataract formation in both in vivo and in vitro models [68,69]. Similarly, in GSH-synthesis knockout (LEGSKO) mice, reduced GSH levels in the lens result in extensive aggregation of oxidized proteins and nuclear cataract formation [70]. Interestingly, research by Wei and co-workers demonstrated that aging-related truncation of the enzyme γ-glutamyl-cysteine ligase catalytic subunit (GCLC), essential for GSH production, accelerates cataract formation, but suppression of this truncation preserves GSH levels and delays cataract development in aging lenses [10].

The GSH antioxidant system is further supported by the enzymatic activities of glutathione peroxidase (GPx), GR and glutaredoxin (Grx). GPx enzymes are essential for maintaining redox balance by reducing H_2_O_2_ and organic hydroperoxides to water and alcohols, using reduced GSH as a cofactor [71]. Although studies on GPx activity in diabetic cataracts have produced mixed results [72,73,74], research has shown that reduced levels of Grx, a key antioxidant protein involved in facilitating GSH-dependent disulfide redox reactions, may contribute to cataract formation [75]. Grx plays a multifaceted role, controlling the cell cycle through the p53/p21/p16 signaling pathway and protecting against oxidative stress by modulating the Akt kinase-forkhead box O1 (Akt-FoxO1), c-Jun N-terminal kinase (JNK), and nuclear factor kappa-light-chain-enhancer of activated B-cells’ (NF-κB) signaling pathways [76,77,78]. In this context, a deficiency in Grx has been associated with increased susceptibility to oxidative stress, particularly from ultraviolet B (UVB) radiation [79]. Moreover, Fan and colleagues found that Grx activity gradually declines with age, and its expression is significantly reduced in the anterior capsule membrane of patients with ARC, corresponding with elevated phosphorylation of extracellular-signal regulated kinase (ERK) [75].

#### 3.1.2. The Thioredoxin System

Another crucial endogenous antioxidant defense system in the lens is the thioredoxin (Trx) system, composed of Trx, thioredoxin reductase (TrxR), peroxiredoxins (Prdx), and methionine sulfoxide reductases (Msrs) [80,81]. This enzymatic system reduces disulfide bonds between proteins, preserving the proteins in a reduced thiol state and maintaining their functionality [82]. Trx exists in two forms, cytosolic Trx-1 and mitochondrial Trx-2, with its activity being regulated by thioredoxin-binding protein-2 (TBP-2) [83]. Hu et al. have shown that Trx-1 and TBP-2 play a role in regulating autophagy induced by oxidative stress, highlighting the protective role of Trx-1 in autophagic processes within human LECs [80]. The importance of autophagy in maintaining lens cell function is well-documented. Failure in autophagy can reduce the ability of the lens to respond to environmental stress or result in abnormal lens development, contributing to cataract formation [84,85]. Moreover, aging not only reduces Grx activity but also decreases Trx activity, potentially increasing the risk of cataracts in the elderly [86].

Prdxs are also essential for regenerating oxidized membrane phospholipids and maintaining ROS balance. Among the six Prdxs, Prdx6 has the highest expression in the lens [87]. This enzyme helps maintain cellular homeostasis and membrane integrity by regulating intracellular phospholipid turnover [88]. Hyperoxidized Prdx6 expression in LECs increases following exposure to UVB radiation [89]. The inactivation of hyperoxidized Prdx6 can lead to elevated ROS production and accelerated cell death in the lens [89]. Kubo and colleagues observed that Prdx6 expression rises postnatally in murine lenses, peaks at six months, and declines with aging [90]. Additionally, research in LEGSKO mice has shown that aged and cataractous lenses exhibit reduced and impaired Prdx6 redox activity compared to younger lenses [91]. Using human lens epithelial cells (HLECs) and Prdx6-deficient cells, Chhunchha et al. demonstrated that curcumin protects cells by upregulating Prdx6 transcription via activation of specificity protein 1 (Sp1), safeguarding against proapoptotic stimuli [92].

**Figure 1:**
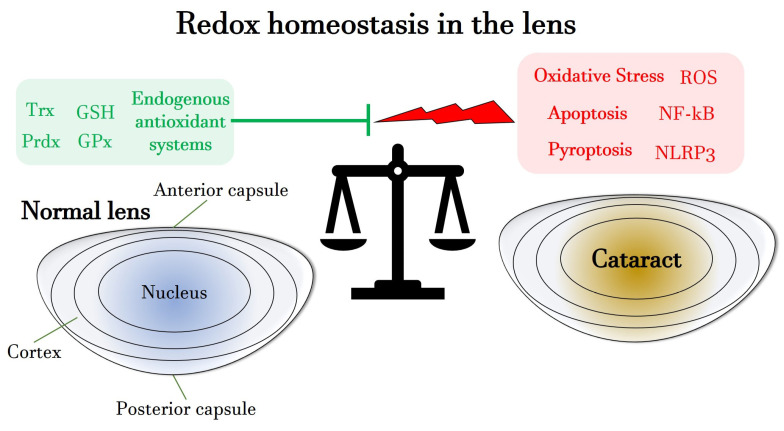
Schematic overview of the redox homeostasis in the lens, highlighting the role of endogenous antioxidants as well as of pro-oxidative, pro-inflammatory and pro-apoptotic agents, responsible for favoring processes of cataractogenesis. GSH: glutathione; GPx: glutathione peroxidase; NF-kB: nuclear factor ‘kappa-light-chain-enhancer’ of activated B-cells; NLRP3: NOD-like receptor protein 3; Prdx: peroxiredoxin; ROS: reactive oxygen species; Trx: thioredoxin.

Figure 1 summarizes the main characteristics of the redox homeostasis in the lens, focusing on the role of endogenous antioxidant systems, such as GSH and Trx.

## 4. Redox Pathomechanisms in Cataract Formation

As the lens ages, a variety of biological, biochemical, and physiological changes occur, leading to damage in lens proteins and ultimately the development of cataracts [93,94]. Extensive research has highlighted the key role of oxidative stress in age-related cataracts (ARCs), with an excess of ROS being a primary contributing factor [95]. Aging significantly reduces antioxidant levels in the lens, increasing oxidative stress and resulting in severe damage to lens proteins, lipids, and DNA [91,96,97]. Such damage is particularly evident in the LECs of individuals with cataracts [98].

Endogenous ROS in LECs can originate from various cellular compartments, including the endoplasmic reticulum (ER), peroxisomes, cellular membranes, and, most notably, mitochondria, where substantial ROS production occurs within the electron transport chain [99,100,101]. Additionally, external factors, such as sunlight exposure, smoking, and heavy metals, exacerbate oxidative damage to the lens [102,103,104]. Several pro-oxidative enzymes, including nicotinamide adenine dinucleotide phosphate oxidase (NOX), cytochrome P450 (CYP450), and xanthine oxidase (XO), have been identified in the lens and contribute to elevated ROS levels, which are associated with lens opacification [105,106,107]. In murine LECs, Das et al. demonstrated that the expression of NOX4 is regulated by transforming growth factor β (TGFβ). The interaction between TGFβ and NOX4-induced ROS production has been implicated in lens epithelial-to-mesenchymal transition (EMT), a process associated with fibrotic cataract [107].

In the following sections, we will explore the key molecular pathways involved in oxidative stress-induced lens damage, highlighting recent research that links redox imbalance with the onset of cataracts during aging, hypertension, and hyperglycemia.

### 4.1. Disruption of the Redox Homeostasis in Age-Related Cataracts

#### 4.1.1. Role of Mitophagy in Cataract Formation

As the lens ages, cellular metabolic functions progressively decline. Real-time bioenergetic profiling of HLECs, conducted using the Seahorse XF96, has demonstrated a reduction in mitochondrial function with aging [108]. Mitochondria are key contributors to ROS generation and the establishment of oxidative stress. Their mitochondrial DNA (mtDNA) is particularly susceptible to oxidative damage [109]. Excessive ROS production can also lead to the release of mitochondrial cytochrome c, which activates the apoptosis cascade [110]. In the lens, mitochondria are primarily found in LECs and early differentiating cortical lens fibers. A study has reported that mtDNA damage in peripheral blood mononuclear cells is significantly higher in patients with ARCs compared to healthy controls [97]. Importantly, LECs undergo mitophagy, the selective autophagy of damaged mitochondria, which prevents excessive ROS accumulation [15]. In particular, exposure of LECs to H_2_O_2_-induced oxidative stress leads to an increase in parkin levels, a key protein in the mitophagy process. This increase promotes the translocation of depolarized or damaged mitochondria, facilitating their removal through the p62/SQSTM1 and ubiquitin ligase pathways [111]. The parkin-mediated clearance of damaged mitochondria may play a critical role in maintaining lens homeostasis by regulating redox levels [111]. Additionally, Wu et al. have demonstrated that, under oxidative stress, glutathione-S-transferase P1 (GSTP1) is a novel substrate of parkin. Parkin promotes GSTP1 degradation via the ubiquitin–proteasome system and mitophagy, thereby compromising the anti-apoptotic function of GSTP1. This mechanism may offer potential therapeutic targets for treating ARC [18].

#### 4.1.2. Endoplasmic Reticulum Stress During Cataract Development

Mitochondria and the ER serve as primary intracellular calcium storage sites. Disruptions in calcium ion homeostasis are intricately linked to mitochondria-induced apoptosis and ER dysfunction [112]. Under normal conditions, the ER is crucial for proper protein folding, safeguarding the cell from stress caused by the accumulation of defective or unfolded proteins [113]. However, the ER itself generates ROS as a result of proteotoxic challenges [114]. When the ER senses an accumulation of misfolded proteins, it activates the unfolded protein response (UPR), an adaptive pathway designed to maintain ER proteostasis and ensure cell survival [115]. However, in cases of prolonged ER stress, the UPR shifts from promoting survival to initiating cell death [116]. This persistent ER stress can generate ROS through UPR-related mechanisms, triggering apoptosis. This process involves the release of calcium from the ER into the cytoplasm, which activates calcium-dependent proteases that cleave essential enzymes and proteins, ultimately impairing normal lens function [117]. Selenium, an essential trace element, is crucial for maintaining cellular health [118]. However, at supra-nutritional levels (>1 μM), selenium becomes a highly toxic pro-oxidant. High doses of selenite, a selenium-containing compound, bind to microtubule proteins, such as tubulin, via disulfide bridges, causing significant conformational changes and inducing ER stress in the lens. Palsamy et al. demonstrated that sodium selenite treatment in HLECs activates a cascade of events, including UPR activation, ER calcium release, and ROS overproduction, eventually leading to cataract formation [119].

#### 4.1.3. Loss or Dysfunction of Epithelial Gap Junctions Contributing to Cataract Formation

Intercellular gap junction channels enable the exchange of metabolites, ions, and fluids between LECs and fiber cells, which is essential for maintaining lens homeostasis, growth, and transparency [120,121]. Notably, an age-dependent decrease in gap junction coupling has been observed in adult lenses, primarily attributed to oxidative damage that degrades connexin proteins. These proteins are critical components of epithelial gap junctions, and their degradation disrupts intracellular homeostasis, potentially contributing to ARNC [122]. The lens contains three types of connexins: α1 (Cx43), α3 (Cx46), and α8 (Cx50), encoded by the genes Gja1, Gja3, and Gja8, respectively [123,124]. In mice, Cx43 is expressed not only in LECs but also in differentiating fiber cells. Cx46, however, is predominantly localized in mature fiber cells. Additionally, Cx50 is significantly expressed in LECs [125,126]. In humans, Cx43 is primarily found in LECs, while Cx46 is concentrated in lens fiber cells. In contrast, Cx50 is expressed in both LECs and fiber cells [120]. Connexin hemichannels, when opened, can release molecules that contribute to inflammatory responses. Blocking these channels has been shown to reduce inflammation and mitigate damage to lens tissues, with their activity regulated by phosphorylation and dephosphorylation [127,128]. Gap junctions help sustain lens homeostasis by facilitating lens microcirculation. Under normal conditions, this microcirculation is supported by Na^+^/K^+^ ATPase, Na^+^/Ca^2+^ exchanger, and Ca^2+^ ATPase on LECs, which transport sodium and calcium out of the lens [129,130].

A missense mutation in Cx50 affects amino acid residue 47 (Cx50D47A in mice and Cx50D47N in humans) [126]. A study by Berthoud and colleagues explored a missense mutation in connexin50 (Cx50), a lens gap junction protein affecting amino acid residue 47. This mutation induces ER stress, which in turn activates the PERK-ATF4 pathway. The activation of this pathway potentially exacerbates lens pathology by promoting the expression of anti-apoptotic factors, thus influencing cell survival and contributing to cataractogenesis [131]. Another study by Berthoud et al. found significantly elevated intracellular calcium levels in Cx50D47A lenses, where decreased connexin expression and gap junctional coupling disrupted lens circulation, increasing hydrostatic pressure gradients and calcium ion concentrations [132]. Additionally, post-translational modifications, like proteolysis, ubiquitination, and phosphorylation, can affect lens microcirculation and may be linked to biomineralization in the lens, such as the formation of calcium oxalate or calcium carbonate crystals observed in cataracts [133,134]. Previous studies in rodent lenses show lower levels of age-related connexin hemichannel truncations in younger lenses, suggesting that connexin modifications depend on age [94,135]. The importance of Cx43 in maintaining lens transparency was highlighted in rodent models, where inhibiting Cx43-mediated coupling in LECs disrupted lens physiology, indicating its importance in lens health [128,136]. Additionally, Shi and colleagues proposed that connexin hemichannels may protect lens fiber cells from oxidative damage by facilitating the intake of reductants, like GSH, from the vitreous humor [64]. In Cx46-knockout mice, the GSH concentration in the lens center is reduced, a finding not observed in Cx50-knockout mice [137]. However, Jara et al. suggested that connexin-knockout cataracts are primarily driven by impaired intercellular calcium circulation rather than GSH passage, implicating calcium dysregulation in cataract formation [138]. In conclusion, the declining function of connexins with age is likely to be a key factor contributing to cataract development.

### 4.2. Oxidative Stress and Cataractogenesis in Hypertension and Diabetes

Systemic hypertension is a risk determinant for the formation of cataracts. Although the influence of antihypertensive drugs on cataractogenesis is substantially debated [139,140,141,142], existing research suggests a link between hypertension, oxidative stress, and cataract development [30,143,144,145,146,147]. Hypertension is associated with overactivation of the renin–angiotensin–aldosterone system (RAAS) [148], which in the lens impairs Na⁺/K⁺ ATPase pump function, promoting cataract formation [143]. In a rodent hypertension model, reduced Ca^2+^ ATPase activity and decreased endogenous antioxidant enzyme levels, such as superoxide dismutase (SOD) and GSH, were observed [149]. A study has recorded a disrupted redox balance and demonstrated that the administration of RAAS inhibitors can reduce oxidative stress and prevent further progression of cataractogenesis [31].

In addition to hypertension, diabetes is also a significant contributor to cataract formation. Chronic hyperglycemia activates the polyol pathway in the lens, leading to excess sorbitol production via aldose reductase, causing osmotic stress, ROS overproduction, and ultimately apoptosis of LECs [32,33,34,35,36]. Like hypertension, chronic hyperglycemia also activates the RAAS [150,151]. In a rodent diabetes model, Ishigooka et al. found a positive correlation between angiotensin-converting enzyme (ACE) levels and oxidative stress markers, such as NOX1, NOX4, and inducible nitric oxide synthase (iNOS), and a negative correlation between ACE and SOD, highlighting the connection between RAAS, oxidative stress, and cataract formation [152]. Shree and colleagues demonstrated that pharmacological inhibition of RAAS overactivation in a rat diabetes model restored the antioxidant level and delayed the onset of cataract formation [153].

**Figure 2:**
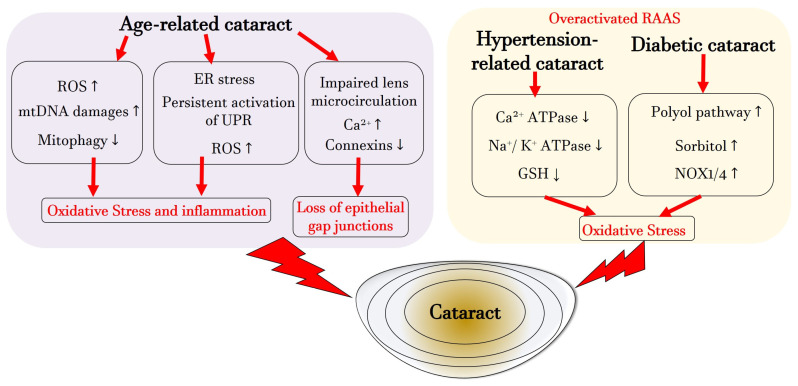
Illustration of the central pathogenetic pathways during cataracto-genesis associated with aging, systemic hypertension or diabetes. ATP: adenosintriphosphat; Ca^2+^: calcium; ER: endoplasmic reticulum; GSH: glutathione; K^+^: potassium; mtDNA: mitochondrial DNA; Na^+^: sodium; NOX: nicotinamide adenine dinucleotide phosphate oxidase; ROS: reactive oxygen species; UPR: unfolded protein response. Upward arrows indicate upregulation or increased activity or increased concentration, whereas downward arrows indicate downregulation or decreased activity or decreased concentration.

Figure 2 illustrates the primary mechanisms leading to cataract formation during aging, hypertension, and diabetes. It emphasizes the role of mitochondria, the ER, loss of gap junctions in ARC, and the significance of the RAAS, ion channels, and NOX in both hypertension and hyperglycemia, collectively identifying oxidative stress as a central pathogenic factor in cataract formation.

### 4.3. Lens Epithelial Cell Death During Cataract Formation

#### 4.3.1. Apoptosis

Apoptosis of HLECs is a hallmark of cataract progression. Peng et al. found that p-coumaric acid (p-CA) mitigates H_2_O_2_-induced apoptosis in HLECs by activating mitogen-activated protein kinase (MAPK) signaling pathways [154]. UVB irradiation, a known trigger of oxidative stress, has also been shown to induce apoptosis in HLECs in a time- and dose-dependent manner, characterized by upregulation of the pro-apoptotic Bcl-2-associated X protein (BAX) gene and downregulation of the anti-apoptotic B-cell lymphoma-2 (Bcl-2) gene [155]. In another study, Ji et al. discovered that the protein calmodulin-like 3 protects HLECs from UVB-induced damage by reducing apoptosis, countering ROS production, decreasing caspase-3 and BAX expression, and increasing Bcl-2 expression [156]. Mechanistically, in the absence of stimulation, NF-κB p65 remains inactive in the cytoplasm by binding to its inhibitor IκB. However, external stimuli, like UV radiation, cause phosphorylation and degradation of IκB, allowing NF-κB p65 to enter the nucleus and regulate target genes, including the Bcl-2 family and pro-oxidative agents, iNOS [157,158,159]. iNOS generates nitric oxide (NO), which combines with O_2_^∙−^ to form peroxynitrite (ONOO−), a highly damaging RNS that contributes to significant damage in LECs [160]. Cartilage acidic protein 1 (CRTAC1), a marker distinguishing chondrocytes from osteoblasts and mesenchymal stem cells, has two isoforms, CRTAC1-A and CRTAC1-B [161]. Sun et al. identified CRTAC1 as a potential NF-κB target gene in UVB-treated HLECs. Overexpression of CRTAC1 promoted ROS generation and induced apoptosis via activation of the p38 signaling pathway [162]. Additionally, sodium-dependent ascorbic acid transporter-2 (SVCT2) protects cells from oxidative stress by regulating ascorbic acid uptake [163]. Guo et al. showed that UVB-induced ROS activated the NF-κB pathway, leading to SVCT2 downregulation in HLECs. This reduced ascorbic acid uptake, promoting ROS accumulation and triggering apoptosis [61]. Ma et al. found that Klotho, an anti-aging protein, mitigates diabetic cataract progression by enhancing nuclear factor erythroid 2-related factor 2 (Nrf2)-mediated antioxidant defenses and inhibiting NF-κB-mediated inflammation [164]. In diabetic cataracts, elevated expression of transient receptor potential vanilloid 2 (TRPV2) in lens tissue has been observed [165]. Increased TRPV2, driven by elevated ROS, triggers apoptosis in LECs through calcium overload in a high-glucose environment, implicating TRPV2 as a key ion channel involved in calcium influx [166].

#### 4.3.2. Pyroptosis

Pyroptosis is a form of programmed cell death characterized by rapid plasma membrane rupture, leading to the release of cellular components and inflammatory mediators, such as IL-1β and IL-18 [167]. This pathway has gained attention as a significant mechanism contributing to ocular diseases, including cataracts, providing new insights into the processes that lead to lens damage. During pyroptosis, the affected cell undergoes chromatin condensation, DNA fragmentation, and membrane disruption [165]. Ultrastructural studies of ARPE-19 and retinal stem cells have shown cytoplasmic swelling, mitochondrial dysfunction, and autophagosome-like structures [168,169]. The inflammasome, a cytosolic signaling complex, is central to triggering inflammation and pyroptosis [170]. Excess ROS can activate the NLRP3 inflammasome and caspase-1 (CASP1), as well as NF-κB, leading to the production of IL-1β and IL-18, pore formation in the plasma membrane, and subsequent cell death [171]. Wang et al. demonstrated in HLE-B3 cells that short-wave blue light exposure induces cell death via pyroptosis, which can be reversed with a CASP1 inhibitor. This blue light was found to activate pyroptosis through the gasdermin D signaling pathway, offering potential targets for cataract prevention [172]. Intriguingly, in a UVB-induced cell damage model, Sun et al. found that cataract patients exhibited significantly elevated levels of pyroptosis markers. They also discovered that downregulating CRTAC1 reversed UVB-induced pyroptosis, while upregulating CRTAC1 promoted pyroptosis in HLECs [173]. These findings further underscore the role of pyroptosis in cataractogenesis and highlight its potential as a therapeutic target for preventing cataracts.

#### 4.3.3. Ferroptosis and Lipid Peroxidation

Cellular membranes, rich in polyunsaturated fatty acids (PUFAs), are highly vulnerable to ROS-induced damage, particularly through lipid peroxidation [174]. Free radicals, such as hydroxyl radicals (•OH), initiate lipid peroxidation by abstracting electrons from PUFAs, forming peroxyl radicals (ROO•). These highly reactive ROO• propagate autocatalytic chain reactions, leading to the generation of hydroperoxides, a key hallmark of ferroptosis [175]. Ferroptosis is a specific form of regulated cell death. Wei et al. found that aged and cataractous human lenses exhibit more ferroptotic markers than any other organ, including indicators of lipid peroxidation, impaired GPx activity, disrupted GSH homeostasis, and an accumulation of redox-active iron [176]. MDA, a product of PUFA peroxidation, is notably elevated in diabetic cataract lenses due to increased oxidant production from glucose oxidation under hyperglycemic conditions [177].

Thus, ferroptosis is fundamentally mediated by ROS, which triggers the release of phospholipids that signal programmed cell death [178]. ROS initiate lipid peroxidation chain reactions, producing reactive aldehydes and peroxides that can also activate apoptosis and autophagy pathways [81,174]. Several studies suggest that lipid peroxidation products can interact with membrane receptors and transcription factors, triggering both intrinsic and extrinsic apoptotic pathways [179,180]. Park et al. demonstrated erastin-induced ROS activate autophagy in ferroptosis, where ROS-induced autophagy regulates ferritin degradation and the expression of transferrin receptor 1 (TfR1) [181]. Furthermore, Dong and colleagues found that FUN14 Domain Containing 1 (FUNDC1) reduces phosphorylation of the PI3K/Akt/mTOR pathway under oxidative stress in SRA01/04 cells, and its deficiency limits apoptosis and autophagy by inhibiting this pathway [182].

LECs, located on the outer surface of the lens, are the first cells exposed to environmental stressors. Their high metabolic rate makes them susceptible to oxidative damage, with lipid oxidation in the lens epithelium being one of the earliest processes in UV-induced lens damage [183]. The lens employs GPx enzymes to combat lipid peroxidation—GPx4 is the only seleno-peroxidase that detoxifies lipid peroxides, while GPx1 detoxifies H_2_O_2_ [184]. Yu et al. showed that melatonin inhibits ferroptosis by activating the sirtuin 6 (SIRT6)/phosphorylated Nrf2 (p-Nrf2)/GPx4 and SIRT6/CoA4/ferritin heavy chain 1 (FTH1) pathways. This action neutralized lipid peroxidation toxicity, reducing ferroptotic stress and preventing UVB-induced cataract formation in rats [185].

**Figure 3:**
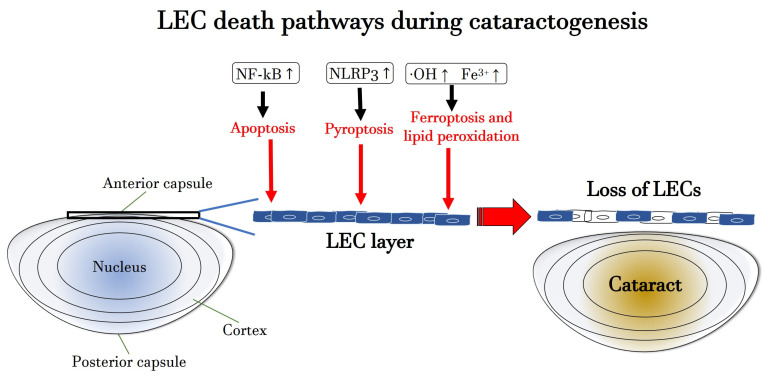
Scheme of the molecular mechanisms of apoptosis, pyroptosis and ferroptosis, leading to LEC death during cataracto-genesis. Fe^3+^: ferric iron; •OH: hydroxyl radical; LEC: lens epithelial cell; NF-kB: nuclear factor ‘kappa-light-chain-enhancer’ of activated B-cells; NLRP3: NOD-like receptor protein 3. Upward arrows indicate upregulation or increased activity or increased concentration.

Figure 3 illustrates the molecular mechanisms of apoptosis, pyroptosis and ferroptosis, leading to LEC death during cataractogenesis.

## 5. Antioxidant Strategies for Preventing Formation and Progression of Cataracts

Currently, cataract surgery, involving the extraction of the cataractous lens and implantation of an intraocular lens (IOL), provides immediate and satisfactory visual recovery for patients [186]. However, the overall prevalence of cataracts remains high and largely unchanged [187]. As a result, there is significant research interest in developing innovative non-surgical treatment strategies aimed at inhibiting or slowing cataract formation. In this context, antioxidants, such as GSH, polyphenols, and specific vitamins, have been explored as potential supplementary treatments. This section provides an updated overview of the challenges and opportunities in developing novel antioxidant-based strategies targeting Nrf2, GSH, and the RAAS to combat cataractogenesis

### 5.1. Nrf2 Activators

Under physiological conditions, the body’s antioxidant defense system maintains a balance between ROS generation and elimination [49]. This system plays a crucial role in preventing oxidative damage, utilizing sophisticated mechanisms to neutralize ROS [56]. The regulation of antioxidant enzyme gene expression is primarily controlled by the nuclear transcription factor Nrf2 [188,189]. This biological process leads to a synchronized increase in the expression of phase II antioxidant genes, such as glutathione S-transferase (GSTπ), catalase (CAT), GPx, HO-1, glutamate–cysteine ligase subunits (GCLC and GCLM), NAD(P)H, quinone oxidoreductase 1 (NQO-1), and Prdxs. However, when the regulation of these protective antioxidants is disrupted, which is commonly seen with aging, cellular function becomes impaired, leading to an increase in oxidative stress and cell death due to excessive ROS production [190,191,192,193,194]. Under normal conditions, Nrf2 is kept inactive by binding to Kelch-like ECH-associated protein 1 (Keap1), which facilitates its ubiquitination and subsequent degradation by the proteasome. When oxidative stress occurs, specific cysteine residues in Keap1, such as Cys-151, Cys-273, Cys-288, Cys-297, and Cys-257, become oxidized. This oxidation triggers the release of Nrf2 from Keap1, where it initiates the transcription of protective antioxidant genes [195]. Research by Chhunchha et al. demonstrated that the FDA-approved drug hydralazine (Hyd) reactivates the Nrf2/ARE pathway in both in vitro and in vivo models. Their work on mouse and human LECs showed that Hyd reduced carbonyl levels, decreased ROS production, and reduced 4-HNE/MDA adducts, providing cyto-protection and delaying lens opacity caused by aging and oxidative stress [196].

Enzymatic antioxidants, like SOD, CAT and GPx [197], as well as non-enzymatic antioxidants, such as vitamin A and GSH, play a pivotal role in defending against oxidative stress [188,198]. These antioxidants either neutralize or scavenge reactive species or interrupt oxidative chain reactions, minimizing oxidative damage [199]. SOD, which is found in mitochondria, the cytosol, and the extracellular matrix, catalyzes the conversion of superoxide radicals into oxygen and H_2_O_2_ [29,37,56,200]. CAT then decomposes H_2_O_2_ into water and molecular oxygen, reducing oxidative stress and preserving mitochondrial structure by enhancing mitochondrial membrane potential (Δψm). This action has anti-apoptotic effects, aiding in cell replication and wound healing [201,202]. In the Trx antioxidant system, Trx and Trx reductase (TrxR) facilitate NADPH-dependent reduction of disulfides in oxidized Trx, restoring it to its active form [203].

Several studies have highlighted the crucial role of enzymatic antioxidants in protecting the lens from oxidative stress, a major contributor to cataract development. In an in vitro study, Zheng et al. found that resveratrol, a known Nrf2 activator, reduced H_2_O_2_-induced cell apoptosis and ROS accumulation, while also inhibiting the phosphorylation of p38 and JNK. These results suggest that resveratrol protects human LECs (HLE-B3) from oxidative damage, potentially through the activation of antioxidant enzymes, like CAT, SOD-1, and HO-1 [204]. In another in vitro study, Lledó et al. demonstrated that melatonin protected cells from H_2_O_2_ and white LED light-induced death. It reduced ROS generation and enhanced antioxidant capacity by increasing Nrf2 levels and SOD activity [205].

Vitamin A scavenges ROO• through electron transfer, preventing lipid peroxidation [206], while vitamin E, a fat-soluble antioxidant, shields PUFAs in membranes from oxidation. Vitamin E also regulates ROS levels and modulates signal transduction pathways [207]. Coenzyme Q10 (CoQ10), the only lipophilic antioxidant involved in mitochondrial respiration, protects against oxidative damage caused by lipid peroxides and promotes mitochondrial biogenesis [208,209]. Ophthalmological and biochemical studies have demonstrated that CoQ10, particularly when administered alone or encapsulated in negatively charged liposomes, exhibits superior efficacy in slowing the progression of cataracts. Furthermore, these formulations have been found to enhance the levels of soluble proteins in the lens and increase the overall antioxidant capacity [210].

Natural antioxidants, such as curcumin, ascorbic acid, and vitamin E, have also been identified as potential therapies for cataract prevention. Curcumin, a potent free radical scavenger and inhibitor of NF-κB, protects against cataract formation due to various factors, including hyperglycemia and hyper-galactosemia [211]. Emerging evidence indicates that curcumin activates the Nrf2 pathway through multiple mechanisms, inhibiting Keap1, modulating upstream Nrf2 regulators, affecting Nrf2 gene expression, and promoting Nrf2 nuclear translocation. Together, these actions contribute to its therapeutic effects [212]. In both in vivo and in vitro studies, Shin et al. revealed that curcumin binds to Keap1 at Cys-151, highlighting this residue as a critical target for Nrf2 stabilization by curcumin by preventing its ubiquitination and degradation [213]. The release of Nrf2 from the Nrf2-Keap1 complex is essential for triggering the Nrf2/ARE pathway, which increases the production of antioxidant enzymes [214]. Recently, an in vitro study by Cao et al. suggested that curcumin may protect the intestinal barrier and mitochondria from oxidative stress by activating the AMP-activated protein kinase (AMPK) pathway [215].

Ascorbic acid acts as a natural defense against UV-induced oxidative damage in the lens. It also helps regenerate vitamin E and GSH, enhancing the antioxidant capacity of the lens. The decline in ascorbic acid levels with age correlates with the severity of cataracts [216]. A review further emphasized the role of ascorbate as a free radical scavenger, highlighting its ability to activate intracellular antioxidant systems and influence pathways like NFκB/TNFα and apoptosis. Moreover, ascorbate promotes the synthesis and activation of antioxidant enzymes, like SOD, CAT, and GPx, and it enhances transcription factors, like Nrf2, redox factor-1 (Ref-1) and activator protein 1 (AP-1), which regulate antioxidant gene expression [217].

Taken together, the utilization of natural antioxidants presents a promising avenue for cataract prevention and management, offering a safe and accessible approach. These compounds, with their diverse mechanisms of action, hold potential in mitigating oxidative stress and its associated damage to the lens.

Unlike most enzymatic antioxidants, non-enzymatic antioxidants are present both within cells and in extracellular fluids, like plasma, tissue fluid, and cerebrospinal fluid, where they serve as the primary defense against oxidative stress [218]. GSH plays a crucial role in oxidative processes within the lens, acting as the primary antioxidant defense [64]. The gradual depletion of GSH with age may contribute to ARC formation by creating conditions that promote protein aggregation and lens opacity [70].

### 5.2. GSH Enhancers

Oxidative stress in LECs plays a critical role in the onset of ARC, emphasizing the importance of lens health in maintaining overall ocular well-being. As a result, there is a significant demand for therapeutic agents capable of preventing oxidative damage to the lens [1].

Thiol-based antioxidants, such as tiopronin (N-(2-mercaptopropionyl)glycine, MPG), N-acetylcysteine amide (NACA), N-acetylcysteine (NAC), and exogenous GSH, have emerged as promising candidates in this context, though their protective effects on LECs are still underexplored [219]. GSH, despite its protective properties, has a short half-life in human plasma (less than 3 min) and faces challenges in entering cells that lack specific transporters [220]. A recent in vitro study by Pfaff et al. demonstrated that treatment with NACA significantly improved cell viability in B-3 HLECs (ATCC CRL-11421) [221] exposed to tert-butyl hydroperoxide (tBHP), a potent inducer of oxidative stress. This protective effect was associated with reduced ROS levels and increased intracellular GSH concentrations. Moreover, supplementation with exogenous GSH also helped maintain cell viability and further boosted intracellular GSH levels. These findings underscore the dual importance of scavenging ROS and enhancing GSH levels to effectively protect LECs from oxidative damage [219]. These compounds hold great potential for developing therapeutic strategies aimed at mitigating oxidative stress in the lens.

NAC functions as both a direct antioxidant and a precursor to GSH [222]. An intriguing study by Savion et al. on human retinal pigment epithelial (RPE) cells (line ARPE-19) revealed that S-allylmercapto-N-acetylcysteine (ASSNAC), a hydrophobic conjugate of NAC and the active residue of allicin (S-allylmercaptan), enhances cell and tissue permeability. This compound upregulates GSH levels and protects the lens from oxidative stress-induced opacity in animal models, suggesting its potential as a therapeutic agent for preventing oxidative stress-related cataract formation [223]. Additionally, Jain et al. investigated the effect of high glucose concentrations on protein oxidation in cultured lens cells and crystalline protein solutions, finding that NAC significantly reduced protein oxidation. This suggests that NAC, along with vitamin B6, may be beneficial in preventing cataracts in diabetic patients [224]. NACA and N-acetyl-carnosine (NAC-N) are two derivatives with improved bioavailability compared to their precursors, addressing challenges related to topical administration. Promising research has shown that topical NAC-N effectively reduces lens opacity in clinical studies [7].

Clinical trials investigating NAC-N as a 1% eye drop formulation have demonstrated positive results over 6 and 9 months of treatment. Lenses treated with a 1% solution twice daily exhibited reduced opacity and glare compared to baseline, suggesting that NAC-N may offer potential for reversing or slowing cataract progression [225,226,227].

In summary, thiol antioxidants, like NAC, NACA, and exogenous GSH, offer promising potential for protecting LECs from oxidative stress and preventing cataracts. Although pre-clinical findings are encouraging, further research is necessary to fully understand their therapeutic efficacy and improve their bioavailability for clinical applications.

### 5.3. RAAS Modulators

Preclinical studies have shown that modulating the RAAS can play a crucial role in preventing cataract formation. In a rodent model of hypertension, olmesartan, an angiotensin receptor blocker, was found to modulate the ocular RAAS, counteract cataract formation, and reduce oxidative stress, ultimately restoring antioxidant activity [31]. Similarly, in streptozotocin-induced diabetic rats, another angiotensin receptor blocker, candesartan, was shown to decrease oxidative stress and inhibit cataract progression [152].

An in vivo study by Shree et al. demonstrated the effectiveness of various RAAS modulators in delaying lens opacity, further supporting the role of RAAS regulation in cataract prevention [153]. Additionally, another in vivo experiment highlighted the positive effects of enalapril, an ACE inhibitor, in a hypertensive cataract model. Enalapril significantly reduced cataract formation by suppressing the upregulation of ocular RAAS and mitigating the oxidative stress that contributes to cataract development [228].

**Figure 4:**
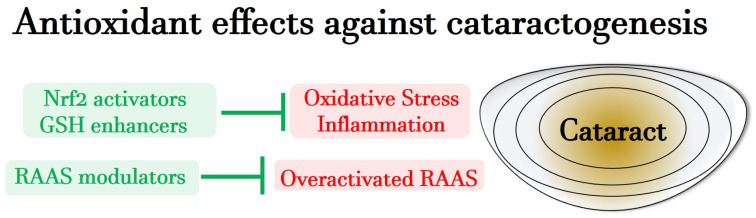
Representation of antioxidative effects, contrasting cataracto-genesis during aging, under hypertension and diabetes. GSH: glutathione; LEC: lens epithelial cell; Nrf2: nuclear factor-erythroid-2-related factor 2; RAAS: renin–angiotensin–aldosterone system.

In summary, recent preclinical studies suggest that RAAS modulators are effective in addressing cataract formation related to hypertension and diabetes by restoring redox homeostasis. These findings underscore a significant link between RAAS, oxidative stress, and cataract formation, presenting a promising therapeutic avenue for cataract management. Figure 4 provides a schematic representation of antioxidant effects contrasting cataractogenesis during aging, under hypertension and diabetes.

## 6. Conclusions

Cataract is a leading cause of irreversible visual loss worldwide. Surgical intervention is the widely most used and effective therapeutic strategy to treat this common ocular disorder. However, the prevalence of cataracts and the frequency of cataract-related blindness are globally increasing, highlighting a substantial deficit in surgical management and indicating a dramatic need to investigate innovative treatment strategies alternative to surgery.

In this context, our review article has shed light on the redox pathomechanisms occurring during cataract formation, offering an updated and comprehensive overview of the main molecular pathways involved.

These processes, closely linked to oxidative stress-induced damage in LECs, are critical to understanding cataract formation. Underscoring the main redox-related pathogenic events in cataract formation is essential for identifying new therapeutic targets and thus finding treatment avenues alternative to surgical intervention.

We presented innovative therapeutic strategies targeting oxidative stress to counteract the formation and progression of cataracts. These include enzymatic and non-enzymatic antioxidants, thiol antioxidants, and natural antioxidants from plants. By highlighting the potential of these molecules, we pave the way for designing novel intervention strategies to combat the onset of cataracts. Further research is warranted to confirm the promising results of these novel potential treatments, and optimization of these approaches may be crucial for addressing the global burden of cataract-related visual impairment and blindness.

## Data Availability

Not applicable.
